# Right-Sided Triorchidism: A Rare Case of Supernumerary Testis in a Young Adult Male

**DOI:** 10.7759/cureus.99710

**Published:** 2025-12-20

**Authors:** Tamer Ewida, Muhammad Abuismaeil, Muhammad Quddus, Ilija Kolevski, Ziauddin Khan

**Affiliations:** 1 Urology Department, Blackpool Teaching Hospitals, Blackpool, GBR

**Keywords:** conservative management, genital ridge duplication, polyorchidism, supernumerary testis, triorchidism, ultrasound

## Abstract

Triorchidism is an exceptionally rare subtype of polyorchidism, defined by the presence of three testes. Most cases are identified incidentally during evaluation for unrelated scrotal symptoms or imaging studies. Although generally benign, this anomaly has diagnostic and management implications because of potential associations with torsion, cryptorchidism, and malignancy. We report a case of a 22-year-old male who presented with intermittent right scrotal discomfort. Examination revealed a normal left testis and two distinct right-sided testes. Scrotal ultrasonography demonstrated two morphologically normal testes with preserved vascularity, homogeneous echotexture, and separate epididymal and ductal structures. Serum tumour markers were within normal limits, and no radiological features suggestive of neoplasia were observed.

The presence of a supernumerary testis is thought to result from segmental duplication of the genital ridge during early gonadal development. When the additional testis is scrotal and radiologically benign, current evidence supports conservative management with routine self-examination and clinical follow-up. Surgical excision is generally reserved for cases with suspicious imaging features or undescended, dysgenetic testes due to the increased risk of malignancy. This report highlights a rare right-sided presentation of triorchidism and reinforces the value of high-resolution ultrasonography in diagnosis. Awareness of this uncommon entity is essential to avoid unnecessary surgical intervention and to preserve fertility potential in otherwise asymptomatic patients.

## Introduction

Polyorchidism refers to the presence of more than two testes and represents an extremely uncommon developmental variation, with triorchidism being the most frequently reported configuration [1]. Fewer than 250 cases have been reported. While many individuals remain asymptomatic, the condition may be associated with torsion, cryptorchidism, infertility, or, in rare cases, malignancy [1]. Embryologically, the condition is thought to result from partial division of the genital ridge during early gonadal differentiation. Recognition of polyorchidism and its benign variants is crucial to avoid unwarranted surgical exploration.

## Case presentation

A 22-year-old male presented with intermittent dull discomfort localised to the right hemiscrotum. He reported no history of trauma, urinary complaints, systemic symptoms, or fertility concerns. Past medical and family histories were unremarkable. On examination, the left testis was normal. The right hemiscrotum contained two distinct, non-tender, ovoid masses, each with a separately palpable vas deferens. No erythema, oedema, masses, or inguinal hernia defects were noted.

Imaging

**Figure 1:**
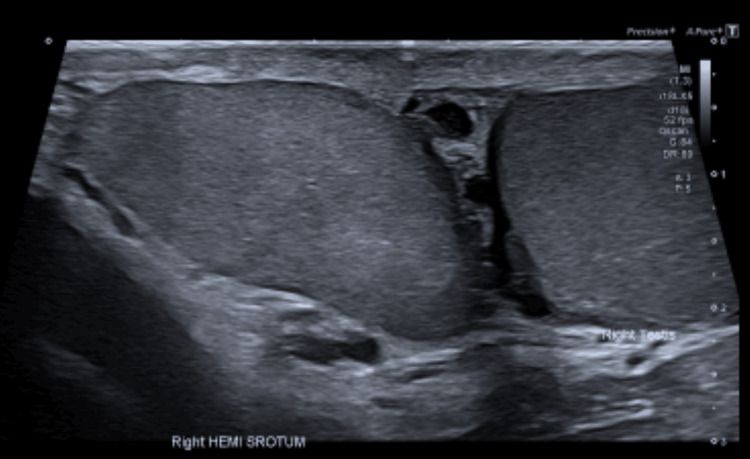
Scrotal ultrasound demonstrating two right-sided testes

High-resolution scrotal ultrasonography identified two morphologically normal right-sided testes with homogeneous echotexture and unobstructed vascular flow (Figure 1). Each testis possessed its own epididymis, measuring approximately 4 × 1.5 × 2.7 cm. Ultrasound remains the diagnostic modality of choice for polyorchidism because of its reliability and accessibility [2]. MRI is reserved for cases in which ductal anatomy or suspicion of neoplasia requires further clarification [3].

Laboratory findings

All serum testicular tumour markers were within normal reference limits, with no biochemical evidence suggestive of malignant activity (Table 1).

**Table 1:** Serum testicular tumour markers

Marker	Value	Reference range
AFP	2.0 kU/L	0–6.6 kU/L
β-hCG	Not detected	Undetectable
LDH	337 IU/L	240–600 IU/L

Diagnosis

**Figure 2:**
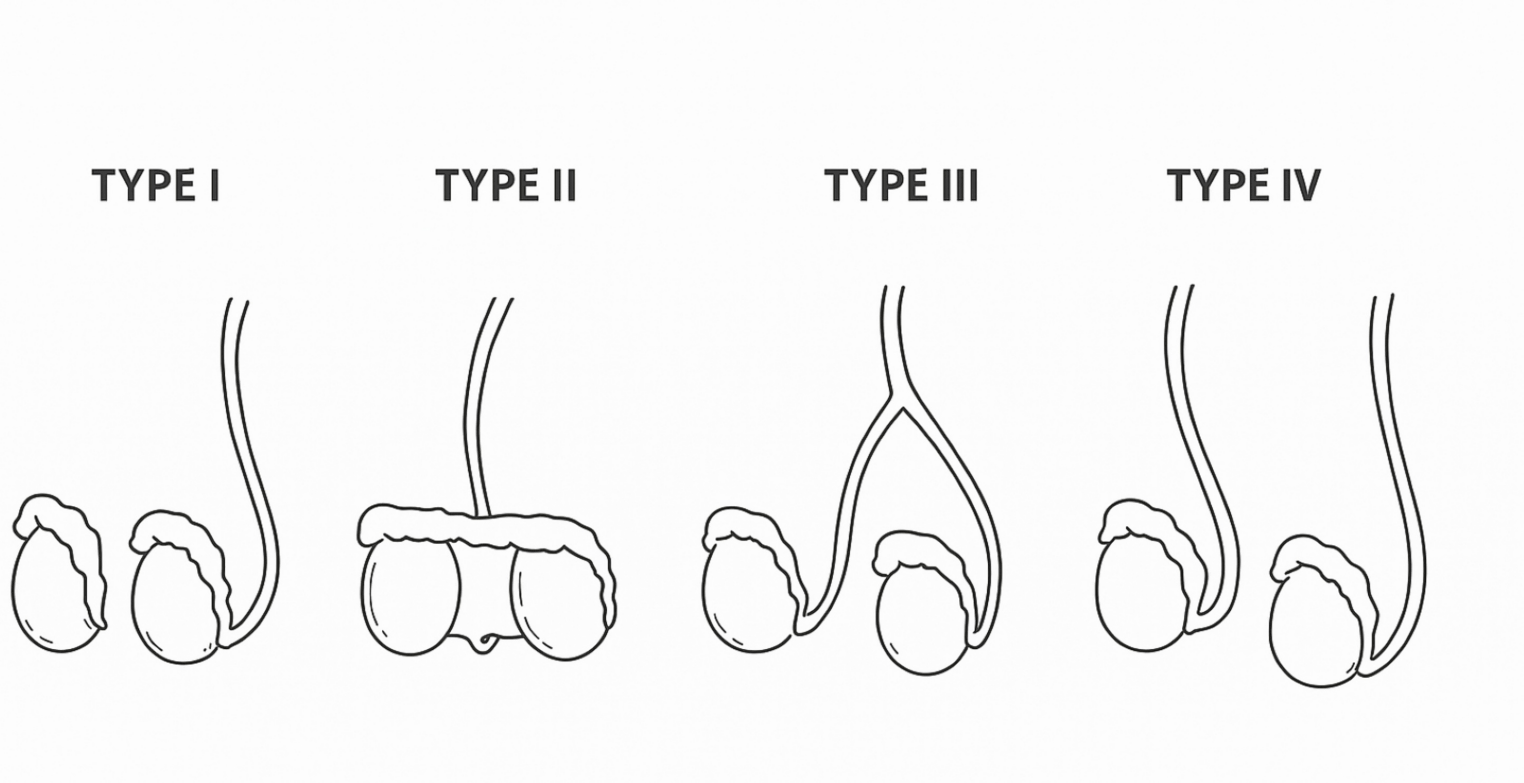
Leung classification of polyorchidism demonstrating four anatomical variants based on epididymal and vasal duplication This schematic is an original illustration created by the authors for the current publication

A diagnosis of right-sided triorchidism consistent with Type IV according to the Leung classification was made, reflecting separation of both epididymal and vasal structures (Figure 2) [4].

Management

Given scrotal location and benign features, conservative management and routine follow-up were advised. The patient was advised to perform regular testicular self-examination and to return if symptoms changed.

## Discussion

Polyorchidism is an uncommon congenital condition, but it remains clinically significant because of its documented associations with torsion, cryptorchidism, infertility, and, on rare occasions, malignancy. A recent systematic review published in 2023 demonstrated that accessory testes located within the scrotum typically exhibit benign functional and structural characteristics, whereas testes positioned in the inguinal canal or abdomen carry a higher risk for adverse outcomes [5]. Most reported cases involve the left testis, making the current right-sided presentation less frequently observed [5].

Embryologic considerations

Testicular development begins during the fourth to seventh weeks of gestation, originating from the genital ridge derived from intermediate mesoderm. Anomalous division or duplication of this ridge, or of its associated Wolffian duct components, can result in the formation of an additional gonadal structure. The extent of division influences whether the accessory testis maintains independent ductal drainage or shares epididymal and vasal pathways with the primary testis, consistent with contemporary anatomic classification criteria [6,7].

Malignancy risk

Although malignant transformation is unusual, pooled data suggest an overall malignancy rate of approximately 4-7% in individuals with supernumerary testes. Nearly all reported malignancies have been associated with non-scrotal or dysgenetic gonads [5,8]. In contrast, accessory testes that are located within the scrotum, demonstrate preserved vascularity and homogeneous parenchyma, and are accompanied by normal tumour markers, are considered low risk and may be managed with surveillance rather than excision [5,8].

Torsion risk

Testicular torsion remains a notable complication. A multi-centre review completed in 2024 documented torsion in approximately 7.2% of reported cases, representing a several-fold increase compared with general adolescent and young adult rates [5]. Proposed contributing factors include increased testicular mobility, altered gubernacular anchoring, and variable epididymal attachment. Patient education on urgent clinical assessment in the event of acute scrotal pain is therefore an integral part of management.

Fertility implications

A reproductive outcomes analysis published in 2022 reported that 50-65% of individuals with polyorchidism demonstrate semen profiles and paternity outcomes comparable with the general population when the accessory gonad is scrotal, well vascularised, and morphologically normal [9]. Conversely, functional impairment is more likely when the supernumerary testis is cryptorchid, previously operated, atrophic, or associated with obstructed epididymal or vasal drainage. These findings support individualised monitoring and highlight the importance of preserving testicular tissue when anatomical and functional features are reassuring [9].

Imaging and management

High-resolution ultrasonography with Doppler remains the preferred initial imaging modality due to its ability to assess vascular integrity, parenchymal uniformity, and ductal relationships. MRI is typically recommended when sonographic findings are ambiguous or when ectopic descent or neoplastic change is suspected [6,7]. In the absence of radiologic abnormality or tumour marker elevation, contemporary recommendations support conservative surveillance and self-examination education. Surgical removal is generally reserved for accessory testes that are extra-scrotal, dysgenetic, or radiologically concerning [5,7,8].

In the present case, the accessory gonad was scrotal, well perfused, sonographically normal, and accompanied by negative tumour markers, all of which align with established criteria supporting non-operative follow-up [10].

## Conclusions

This case represents a rare, right-sided presentation of triorchidism diagnosed using ultrasonography. Conservative management remains the appropriate approach for cases demonstrating benign imaging features and scrotal positioning. Awareness of this entity helps avoid unnecessary intervention and supports preservation of long-term reproductive health.
